# Endometrial tuberculosis compounding polycystic ovary syndrome in a subfertile woman: a case report

**DOI:** 10.1186/s13256-016-0959-7

**Published:** 2016-06-07

**Authors:** Charles Mariara, Angela Koech, Peter Waweru, Alfred Murage

**Affiliations:** 1 AIC Kijabe Hospital, P.O Box 20-00220, Kijabe, Kenya; 2 Aga Khan University Hospital, 3rd Parklands Avenue, Box 30270-00100, Nairobi, Kenya

**Keywords:** Subfertility, Genital tuberculosis, Polycystic ovary syndrome

## Abstract

**Background:**

Asymptomatic female genital tuberculosis can impair tubal and endometrial function and later present as subfertility. A majority of the patients with genital tuberculosis in endemic regions present with subfertility and the delay in presentation, coupled with the potential the disease has in mimicking other gynecological conditions, renders it elusive. In addition to the challenge of diagnosing genital tuberculosis, fertility outcomes after treatment are not impressive. This is particularly so in the background of another confounding subfertility factor to which interventional efforts may initially be directed, at the expense of undiagnosed genital tuberculosis. We therefore present a case of subfertility due to endometrial tuberculosis, but confounded by other subfertility factors notably polycystic ovary syndrome. To the best of our knowledge this case report is the first of its kind in the literature.

**Case presentation:**

This is a case report of a 42-year-old woman of African descent who presented to our fertility clinic with a 10-year history of primary subfertility and amenorrhea of 6 years duration. She was a nurse in a medical ward and had no prior history of tuberculosis. She had undergone a diagnostic laparoscopy 8 years prior which demonstrated dense pelvic adhesions and an impression of tubal factor subfertility was made. At presentation, her gonadal hormone profile and pelvic ultrasound were consistent with polycystic ovary syndrome. A negative response to a progesterone challenge test prompted a hysteroscopic evaluation which revealed endometrial atrophy. Endometrial biopsies confirmed histological features consistent with tuberculosis. Normal endometrial function was not restored despite adequate treatment and her options were limited to surrogacy or adoption.

**Conclusions:**

Genital tuberculosis is elusive in presentation and clinicians should consider it in patients with amenorrhea and/or tubal disease from tuberculosis-endemic regions. Due to the attendant high cost of fertility treatment and associated poor fertility outcomes, it is prudent to explore options to diagnose it early. A routine endometrial biopsy in a patient with subfertility in a tuberculosis-endemic area would be pragmatic. An alternative algorithm in management would be risk stratification prior to endometrial biopsy.

## Background

The exact incidence of female genital tuberculosis (TB) is difficult to ascertain given the quiescent nature of the disease and the varied diagnostic methods. Subfertility might be the only symptom of genital TB and is associated with tubal damage in a majority of cases [1]. Hematogenous spread is thought to be the process by which the tubercle bacilli implant into the mucosa of the fallopian tubes and subsequently affect the endometrium by direct extension [2].

Diagnosis of female genital TB requires a high index of suspicion due to its asymptomatic nature and the high probability of the disease mimicking other gynecological conditions [3]. In addition to the diagnostic challenge, fertility outcomes after treatment are not impressive [4] particularly in the background of another factor causing subfertility. A diagnostic hysteroscopy and endometrial biopsy that are often necessary to arrive at a diagnosis are not without challenges of accessibility and cost in TB-endemic regions. The coexistence of genital TB with other gynecological conditions such as polycystic ovary syndrome (PCOS) leads to a delay in diagnosis and compounds the challenges in treatment of subfertility. The objective of this case report is to highlight the challenges in diagnosis and management of genital TB, particularly in the background of other subfertility factors, and to consider pragmatic approaches in the treatment of subfertility in TB-endemic areas.

## Case presentation

We present a report of a 42-year-old woman of African descent who presented to our fertility clinic with a 10-year history of primary subfertility and amenorrhea of 6 years duration. She was a nurse in a medical ward and had no prior history of TB. She was separated from her husband and intended to use donated sperm for her assisted conception treatment. The timeline of interventions is summarized in Table 1 below.

**Table 1:** Timeline of interventions for our patient

Year	Age (years)	Notable findings
2004	33	Diagnostic laparoscopy revealing dense pelvic adhesions
2006	35	Onset of amenorrhea
2012	41	Gonadal profile: elevated E2, high AMH for age (5.18 ng/ml).
		Progesterone challenge test: no withdrawal bleed.
		Ultrasound: polycystic ovaries, endometrial fluid, ET 3 mm
2013	42	Hysteroscopy and endometrial biopsy: TB endometritis.
2013	42	Started anti-TB treatment
		Completed anti-TB antibiotics. No menses
		Administration of COC: no menses

*AMH* anti-Müllerian hormone, *COC* combined oral contraceptives, *E2* estradiol, *ET* endometrial thickness, *TB* tuberculosis

The initial evaluation for subfertility (in a different institution) included a diagnostic laparoscopy performed 8 years prior. The laparoscopy had revealed dense pelvic adhesions (“a frozen pelvis”), and an impression of tubal factor subfertility was made. It appears that there was no deliberate attempt to make a definitive diagnosis as to the cause of the tubal disease as no tissue samples or swabs were assessed. She had undergone a pelvic ultrasound scan which was reported as normal and had had a negative pap smear. There were neither further details of this prior evaluation nor a detailed summary from her previous gynecologist.

She neither had a history of pelvic pain nor a history suggestive of previous sexually transmitted infections. She did not report any symptoms suggestive of TB, notably fever, night sweats, or weight loss. She weighed 84 kg, was 1.58 meters tall with a body mass index (BMI) of 33. Her blood pressure was 140/90 mmHg and her clinical examination was essentially normal. She had no lymphadenopathy, acne, or hirsutism. Her gonadal hormone profile presented in Table 2 was in keeping with PCOS.

**Table 2:** Gonadal hormone profile

Investigation	Result
17 beta estradiol	3447 (99–452) pmol/L
Total testosterone	5.2 (0.52–2.4) nmol/L
Follicle-stimulating hormone (FSH)	6.6 (1.0–8.8) IU/L
Luteinizing hormone (LH)	38.4 (1–18) IU/L
Prolactin	0.45 (0.09–1.26) nmol/L
Anti-Müllerian hormone	37.00 pmol/L (elevated for age)

The initial high estradiol (E2) levels and the biochemical evidence of PCOS prompted the use of an exogenous progestogen to initiate endometrial shedding prior to baseline ultrasound scan in preparation for an *in vitro* fertilization (IVF) cycle. She received medroxyprogesterone (Provera) 10 mg orally once a day for 5 days but had no withdrawal bleed. Two weeks after administration of medroxyprogesterone, she still reported no withdrawal bleed. A subsequent pelvic ultrasound scan showed normal uterine morphology. Her endometrial thickness was 3 mm with a small amount of endometrial fluid with no focal endometrial pathology demonstrable on ultrasound. Her ovaries had a polycystic appearance with multiple small peripheral follicles. A simple cyst of 4 cm in size was visualized in her right ovary. The ultrasound findings further supported the diagnosis of PCOS.

Endometrial assessment by hysteroscopy was undertaken in view of the presence of endometrial fluid and the negative progesterone challenge test. Hysteroscopy revealed an atrophic endometrium interspersed with areas of thickened endometrium, with no synechiae, and bilaterally obliterated and poorly visualized tubal ostia. Directed endometrial biopsies were taken. Histology showed endometrial tissue with a normal gland to stromal ratio. Numerous granulomas, with epithelioid histiocytes, plasma cells and Langhans giant cells were visualized in the stroma with some areas of necrosis. A Ziehl–Neelsen stain was negative. A conclusion of granulomatous endometritis possibly TB endometritis was made.

Following the diagnosis of TB, further investigations were carried out. A chest X-ray was normal and an HIV test was negative. Her liver function tests, creatinine, and full blood count were all normal. She was referred to a physician for TB treatment and plans for assisted reproductive technology (ART) were deferred. Treatment for TB endometritis was started by use of a 6-month regimen of antitubercular therapy. An initial 2-month intensive phase of four drugs (rifampicin 600 mg once daily, isoniazid 300 mg once daily, pyrazinamide 2000 mg once daily, and ethambutol 1600 mg once daily) was followed by a 4-month course of two drugs (rifampicin 600 mg once daily, isoniazid 300 mg once daily). In addition, a daily dose of pyridoxine 25 mg was administered throughout the 6 months. Antihypertensive treatment was initiated in the second month of antitubercular treatment. She was evaluated after a week of initiation of TB therapy and thereafter every month by the physician. She was compliant on all her medications and experienced no adverse effects. She reported no menses despite completing the TB treatment. The supervising TB physician did not consider it clinically relevant to re-biopsy after a full course of TB therapy.

She maintained her desire for conception and revisited the fertility clinic. A follow-up ultrasound scan showed less evident endometrial fluid compared to the initial pelvic ultrasound scan with an endometrial thickness of 3.7 mm. Her ovaries were still polycystic but the 4 cm right ovarian cyst was no longer present. A decision on a second trial of endometrial stimulation/shedding was made. She received one cycle of low-dose combined oral contraceptive pills but reported no menses. There was no change in endometrial findings on follow-up scan.

A conclusion of endometrial scarring post-endometrial TB was made. Further attempts at endometrial stimulation were discouraged in view of her very high endogenous E2 and complete failure of response to exogenous steroids. The options of surrogacy or adoption were discussed while further ART interventions were discouraged due to the endometrial damage.

## Discussion

The limitations in management of this case were primarily a delay in definitive diagnosis and treatment. This could have had a compounding effect on our patient’s fertility in that not only did tubal and endometrial damage ensue but also allowed an age-related decrease in ovarian function, a factor noted to be the most significant as regards fertility outcomes in IVF [5]. It remains open to discussion if an earlier diagnosis and treatment of the genital TB may have made a difference in her reproductive outcomes following ART. Of note, however, a possible risk factor that the patient had was her profession which potentially could have exposed her to TB.

This case highlights the need for prompt and efficient referrals in patient management. Sufficient details were unavailable upon referral including details of the initial diagnostic laparoscopy that had demonstrated tubal disease. In addition, health care costs were a significant constraint for this patient, which demonstrates the challenges of fertility treatment particularly in resource-limited settings.

Following failure of resumption of endometrial function, the patient went into denial. She continued to visit the fertility clinic occasionally, inquiring about the possibility of new interventions. She remains hopeful that endometrial function will resume prior to menopause. She still desires to carry her own pregnancy and is not keen on surrogacy with her own oocytes. It is important to highlight that patients presenting with subfertility require psychosocial support at every step of management.

## Conclusions

This original case report seeks to inform all clinicians, particularly those in reproductive medicine, that genital TB is a diagnosis that requires a high index of suspicion to diagnose especially when the patient’s history and clinical examination is not suggestive of the disease [3]. Its elusive nature and tendency to mimic other gynecological conditions can lead to delay in diagnosis. Occasionally, some patients present with subfertility and their fertility treatment options become limited particularly those with endometrial damage. Given the elusive nature of genital TB and the high cost of fertility treatment, it is prudent to explore options to diagnose it early. Despite it being difficult to ascertain to what extent a routine endometrial biopsy in a patient with subfertility is beneficial, some fertility centers in TB-endemic areas consider this approach to be pragmatic for all patients presenting with subfertility [6]. An alternative algorithm in management would be risk stratification prior to endometrial biopsy.

## Abbreviations

ART, assisted reproductive technology; E2, estradiol; IVF, *in vitro* fertilization; PCOS, polycystic ovary syndrome; TB, tuberculosis
